# Distinct regulatory machineries underlying divergent chromatin landscapes distinguish innate lymphoid cells from T helper cells

**DOI:** 10.3389/fimmu.2023.1271879

**Published:** 2023-12-01

**Authors:** Yime Zhang, Luni Hu, Guanqun Ren, Yanyu Zeng, Xingyu Zhao, Chao Zhong

**Affiliations:** ^1^ Department of Immunology, School of Basic Medical Sciences, Peking University Health Science Center, Beijing, China; ^2^ Key National Health Commission Laboratory of Medical Immunology, Peking University, Beijing, China; ^3^ Institute of Systems Biomedicine, School of Basic Medical Sciences, Peking University Health Science Center, Beijing, China; ^4^ Beijing Key Laboratory of Tumor Systems Biology, Peking University, Beijing, China

**Keywords:** innate lymphoid cells, T helper cells, transcriptome, chromatin accessibility, transcription factor

## Abstract

Innate lymphoid cells (ILCs), as the innate counterpart of CD4^+^ T helper (Th) cells, play crucial roles in maintaining tissue homeostasis. While the ILC subsets and their corresponding Th subsets demonstrate significant similarities in core programming related to effector function and regulatory mechanisms, their principal distinctions, given their innate and adaptive lymphocyte nature, remain largely unknown. In this study, we have employed an integrative analysis of 294 bulk RNA-sequencing results across all ILC and Th subsets, using scRNA-seq algorithms. Consequently, we identify two genesets that predominantly differentiate ILCs from Th cells, as well as three genesets that distinguish various immune responses. Furthermore, through chromatin accessibility analysis, we find that the ILC geneset tends to rely on specific transcriptional regulation at promoter regions compared with the Th geneset. Additionally, we observe that ILCs and Th cells are under differential transcriptional regulation. For example, ILCs are under stronger regulation by multiple transcription factors, including RORα, GATA3, and NF-κB. Otherwise, Th cells are under stronger regulation by AP-1. Thus, our findings suggest that, despite the acknowledged similarities in effector functions between ILC subsets and corresponding Th subsets, the underlying regulatory machineries still exhibit substantial distinctions. These insights provide a comprehensive understanding of the unique roles played by each cell type during immune responses.

## Introduction

In recent years, innate lymphoid cells (ILCs) have garnered increasing attention owing to their functional parallels with CD4^+^ T helper (Th) cells of the adaptive immune system (1–3). Both ILCs and Th cells play pivotal roles by secreting effector cytokines, sharing similarities in their effector functions and regulatory mechanisms. These shared characteristics lead to the categorization of both cell types into distinct subsets based on the expression of signature effector cytokines and master transcription factors (4–7). For instance, type 1 ILCs (ILC1s) and Th1 cells mainly produce IFN-γ and TNF-α, regulated by the master transcription factor T-bet (8, 9). Likewise, ILC2s and Th2 cells predominantly express IL-5 and IL-13, controlled by the transcription factor GATA3 (10–12). Furthermore, ILC3s, encompassing natural killer receptor expressing (NCR^+^) ILC3s, double-negative (DN) ILC3s, and lymphoid tissue-inducer (LTi) cells, mirror Th17 cells as they primarily secrete IL-22 and IL-17, under the regulation of the transcription factor RORγt (13, 14).

Although the ILC subsets and their corresponding Th subsets share functional and regulatory similarities, it remains imperative to acknowledge their fundamental differences as innate and adaptive lymphocytes, respectively. One notable distinction lies in the role of T-cell receptor (TCR) signaling, which is crucial for the activation, differentiation, and effector functions of Th cells (15). TCR signaling triggers downstream transcription factors like AP-1, NF-AT, and NF-κB (16). In contrast, ILCs, lacking TCR expression, rely on cytokines, neuropeptides, eicosanoids, and other environmental signal for activation (17–21). Additionally, ILCs are characterized by their tissue residency, meaning that their activation and functions are regulated by local environmental cues and they exert crucial regulatory roles in maintaining tissue homeostasis (22, 23). Th cells, however, are primarily circulatory, undergoing initially activated and differentiated in secondary lymphoid organs before migrating to exert effector functions in peripheral tissues (24). This distinction underscores that despite parallels, there exist key functional and regulatory differences between ILCs and Th cells intrinsic to their identities as innate versus adaptive lymphocytes.

The transcriptomic differences between ILC subsets and their corresponding Th cells should, to varying degrees, reflect their functional and regulatory differences. Previous studies have attempted transcriptional comparison between these cell types (25–27). For instance, in a study examining *Nippostrongylus brasiliensis* infection, lung ILC2s and Th2 cells isolated on day 14 were subjected to bulk RNA-sequencing (RNA-seq) analysis. The resulting differentially expressed genes between the two cell types were indeed substantial (25). However, the core programing of lung ILC2 and Th2 cells, including cell-surface receptors, cytokines, and transcription factors, exhibited significant shared properties (26, 28). Furthermore, other studies have demonstrated that differentiated Th2 cells and memory Th2 cells can produce effector cytokines independently of TCR signaling, similar to innate lymphocytes (29, 30). These findings may partially explain the resemblance between lung Th2 cells and ILC2s after *N. brasiliensis* infection. Nevertheless, it remains unclear whether all ILC subsets and their corresponding Th cells exhibit such comparable features under different conditions. Moreover, ILCs primarily reside in local tissues, where they participate in maintaining tissue homeostasis. Conversely, Th cells typically undergo activation and differentiation in secondary lymphoid organs, specializing in cytokine production to facilitate immune responses. Therefore, further investigation into the fundamental difference between ILCs and Th cells is still imperative.

## Results

### Integrated transcriptome analysis uncovers distinct gene expression signatures between ILC and Th subsets

**Figure 1:**
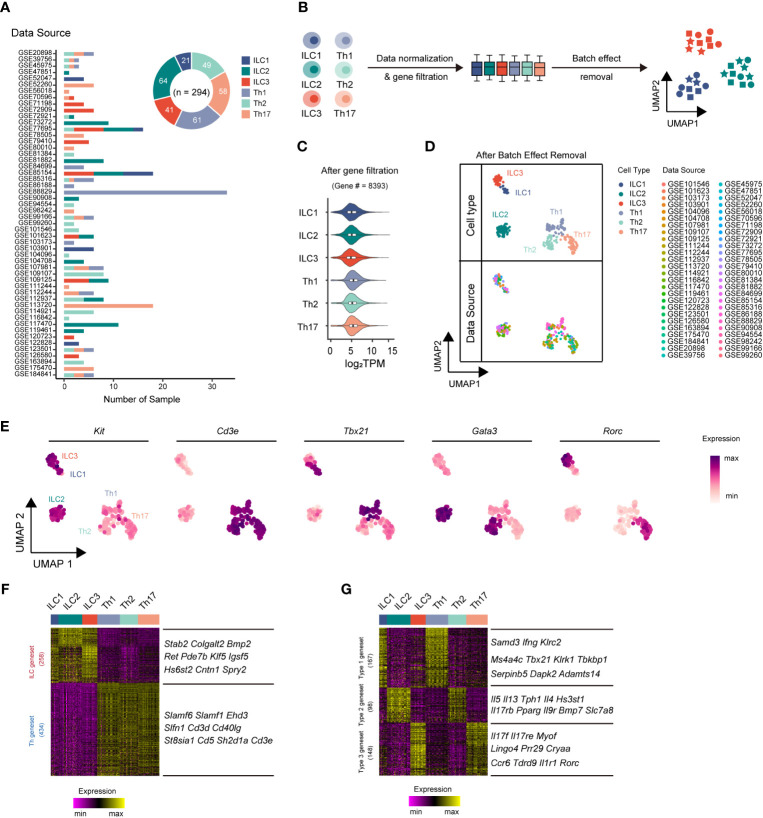
Integrative RNA-seq analysis uncovers principal genesets distinguishing ILC and Th subsets. **(A)** Bar chart showing the sources and numbers of bulk RNA-seq datasets across different ILC and Th subsets, and donut plot showing the numbers of bulk RNA-seq datasets collected for each cell type. **(B)** Schematics of the RNA-seq data preprocessing strategy. RNA-seq datasets of ILCs and Th cells are normalized into TPM, and then low-expression genes are filtered out, followed by batch effect removal using the limma package. **(C)** Violin plot showing average gene expression (log_2_ TPM) in each cell type after low expression gene filtering. **(D)** UMAP plot showing distribution of ILC and Th RNA-seq samples after batch effect removal. Cell types and data sources are annotated. **(E)** Scatter plot showing the expression of *Kit*, *Cd3e*, *Tbx21*, *Gata3*, and *Rorc* across ILC and Th subsets. **(F)** Heatmap showing differentially expressed genes between ILC and Th (log_2_ fold-change > 0.25, P. value < 0.01). The top 10 ILC-specific and Th-specific genes ranked by fold change are listed. **(G)** Heatmap showing conserved genes in ILC and Th subsets of each immune response (log_2_ fold change > 0.25, P. value < 0.01). The top 10 genes with minimum fold change among ILC and Th subset are listed. UMAP, Uniform Manifold Approximation and Projection. TPM, transcripts per million mapped reads.

ILCs and Th cells exhibited parallel effector functions in innate and adaptive immunity, respectively (31). However, the transcriptomic differences between these cell types had remained largely unclear. To comprehensively compare their transcriptomes and minimize variations from a single data source, we performed an integrative analysis leveraging publicly available bulk RNA-sequencing (RNA-seq) datasets. In total, we collected bulk RNA-seq datasets from 52 published studies, comprising 294 samples, including 21 ILC1, 64 ILC2, 41 ILC3, 61 Th1 cell, 49 Th2 cell, and 58 Th17 cell samples (**Figure 1A**, **Supplementary Table 1**). Subsequently, we applied a series of data processing steps to the bulk RNA-seq datasets, including normalization of data, filtration of genes with low expression, and removal of batch effect (**Figure 1B**, **Supplementary Figure 1A**). Through these steps, we identified 8,393 genes with significantly high expression levels (transcripts per million or TPM >10) in at least one ILC or Th subset (expressed in more than 80% of the samples for each respective subset) (**Figure 1C**, **Supplementary Figure 1B**). Following the removal of batch effects, the RNA-seq samples underwent UMAP classification, a commonly used algorithm frequently used for single-cell RNA-seq (scRNA-seq) analysis, based on the gene expression profiles of the samples (**Figure 1D**, **Supplementary Figure 1C**). The UMAP analysis accurately assigned the samples to their respective cell type categories, indicating that the gene expression profiles effectively captured the cellular heterogeneity within the dataset. To further validate the accuracy of the RNA-seq dataset processing, we examined the expression of expected signature genes in ILC and Th subsets. Specifically, all three ILC subsets exhibited enriched expression of the ILC marker *Kit*, whereas the three Th subsets showed specific expression of the T-cell marker *Cd3e* (**Figure 1E**). Moreover, the master transcriptional factors *Tbx21*, *Gata3*, and *Rorc*, associated with different immune cell types, were accurately and highly expressed by their respective ILC or Th subsets (**Figure 1E**).

The successful classification allowed us to further investigate the distinct transcriptional features present in each ILC and Th subset by employing algorithms specifically designed for scRNA-seq analysis. Despite sharing similarities in effector functions, we anticipated significant transcriptional differences between ILC subsets and their corresponding Th subsets, reflecting their inherent differentiation as innate and adaptive immune cells, respectively. To elucidate such transcriptional differences, we conducted paired transcriptome comparisons between ILC subsets and their parallel Th subsets (ILC1 versus Th1, ILC2 versus Th2, and ILC3 versus Th17). This analysis revealed 258 ILC-specific genes and 434 Th-specific genes, referred to as the ILC geneset and Th geneset, respectively (**Figure 1F**, **Supplementary Table 2**). To further verify the reliability of the geneset and eliminate the interference of the tissue environment, we conducted a comparison of the expression levels of the ILC geneset and the Th geneset between ILC and Th subsets originating from the same source (25, 32, 33). As we anticipated, most genes in the ILC geneset exhibit a higher expression level in ILC subsets, whereas most genes in the Th geneset exhibit a higher expression level in Th subsets from the same source (**Supplementary Figure 2**). Overall, the ILC geneset and Th geneset represent fundamental distinctions between ILC and Th cells and are minimally affected by environmental factors.

The different subsets of ILCs, along with their corresponding Th subsets, play distinct roles in immune responses. Therefore, we next conducted a screening process to identify genes that underlay specific immune responses, exhibiting conserved, subset-specific expression patterns in individual ILC subsets and their corresponding Th counterparts, aiming to identify genes underlying specific immune responses. As a result, we defined 167 genes as the type 1 geneset, which included *Ifng* and *Tbx21*, specifically expressed in both ILC1s and Th1 cells. We also defined 98 genes as the type 2 geneset, such as *Il4*, *Il5*, *Il13*, and *Gata3*, which were preferentially upregulated in both ILC2s and Th2 cells. Additionally, we defined 148 genes as the type 3 geneset, like *Il17a*, *Il17f*, *Il22*, and *Rorc*, exhibiting significant increases in both ILC3s and Th17 cells (**Figure 1G**, **Supplementary Table 3**).

Collectively, by conducting an integrated analysis of 294 bulk RNA-seq datasets across ILC and Th subsets using scRNA-seq algorithms, we have successfully identified genesets that highlight the key transcriptional differences between ILCs and Th cells, as well as the variations in immune response programs.

### GO enrichment analysis reveals fundamental functional and regulatory disparities across ILC and Th subsets

**Figure 2:**
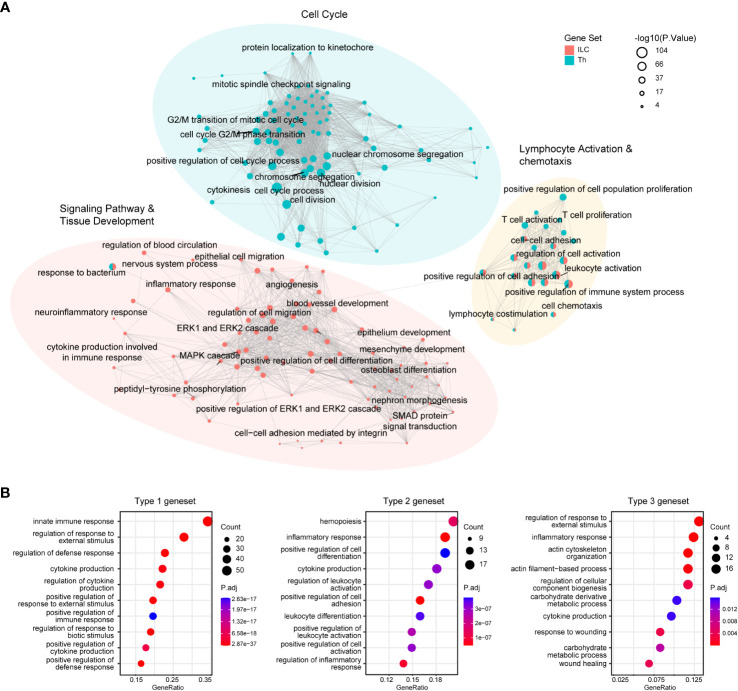
GO enrichment analysis reveals fundamental functional and regulatory differences between ILCs and Th cells. **(A)** GO analysis of the ILC and Th genesets. Enrichment map shows the top100 terms enriched in the ILC or Th genesets. **(B)** Dot plot showing the top10 terms enriched in immune response-specific genesets. GO, gene ontology.

These genesets identified in our analysis provided valuable insights into the fundamental transcriptional characteristics underlying the functional properties of the ILC and Th subsets. To further elucidate the functional differences between ILCs and Th cells, as well as between distinct immune response programs, we performed gene ontology (GO) enrichment analysis on these genesets. The analysis revealed the top 100 enriched GO terms in the ILC and Th genesets, highlighting the significant overrepresentation of pathways related to “lymphocyte activation and chemotaxis” in both ILCs and Th cells. This finding underscored the critical role of these processes in shaping the functional properties of ILCs and Th cells in the immune system (**Figure 2A**, **Supplementary Tables 4**, **5**). To validate this observation, we examined the expression profile of representative genes within these GO terms across different ILC and Th subsets (**Supplementary Figure 3A**). Additionally, GO terms associated with pathways of “cell migration”, “epithelium development”, “mesenchyme development”, “blood vessel development”, and “nervous system process” were specifically enriched in the ILC geneset (**Figure 2A**, **Supplementary Table 4**), consistent with the known role of ILCs as tissue-resident immune cells involved in maintaining tissue homeostasis (22, 23). Specifically, GO terms related to “cytokine production”, “MAPK-ERK pathway” and “SMAD pathway” were also enriched in the ILC geneset, suggesting distinct regulation machineries underlying the immune effects of ILCs compared with Th cells (**Figure 2A**). In contrast, GO terms associated with “cell cycle” were highly enriched in the Th geneset, indicating fundamental disparities between Th cells and ILCs in terms of proliferation (**Figure 2A**, **Supplementary Table 5**). This observation aligned with the knowledge that T cells underwent clonal expansion during activation and differentiation, resulting in a significant increase in cell number (34). Furthermore, the expression profiles of representative genes within the specifically enriched GO terms in the ILC and Th genesets confirmed the functional distinctions between ILCs and Th cells (**Supplementary Figures 3B–D**).

In addition, we examined the enriched GO terms in the three immune response-related genesets. Although there were discernible differences between these immune response programs, it was noted that GO terms associated with cytokine production, defense response, and inflammatory response were consistently enriched across all three genesets (**Figure 2B**, **Supplementary Figures 3E–G**, **Supplementary Tables 6**–**8**). This suggested a similarity in their helper-like functionalities and their contribution to immune responses.

Together, the combined GO enrichment analysis of the predefined ILC and Th genesets, as well as the immune response-related genesets, provides a comprehensive understanding of the functional and regulatory similarities and differences between the different ILC and Th subsets.

### Expression-concordant opening chromatin regions associated with the ILC geneset tend to distribute in close proximity to the transcription start sites

**Figure 3:**
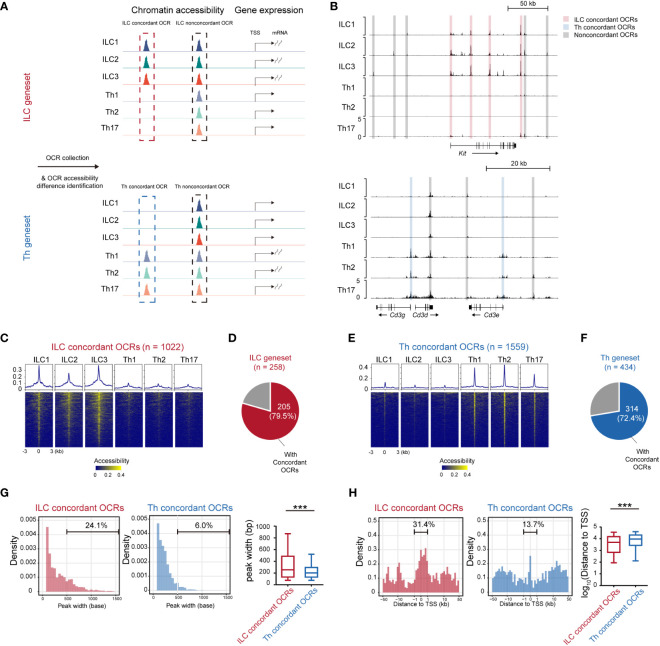
Expression-concordant opening chromatin regions associated with the ILC geneset tend to distribute in close proximity to the transcription start sites. **(A)** Schematics of identifying all OCRs associated with the genes in the ILC and Th genesets. **(B)** DNase-seq tracks at the *Kit* locus and *Cd3g*, *Cd3d*, and *Cd3e* loci in ILC and Th subsets. Expression-concordant OCRs (fold change > 1.5 between ILC and Th, minimum BPM in both repeats of each ILC subset > maximum BPM in both repeats of each Th subset, BPM in all ILC/Th sample > 0.5, red in ILC and blue in Th) and expression-non-concordant OCRs (gray) are signed. **(C)** Heatmap showing chromatin accessibility of ILC concordant OCRs in the ILC and Th subsets. Profile plot illustrates the average chromatin accessibility of corresponding regions. **(D)** Pie chart showing the number and proportion of genes in the ILC geneset with ILC concordant OCRs at their gene loci. **(E)** Heatmap showing chromatin accessibility of Th concordant OCRs in ILC and Th subsets. **(F)** Pie chart showing the number and proportion of genes in the Th geneset with Th concordant OCRs at their gene loci. **(G)** Histogram showing peak size distribution of expression-concordant OCRs in ILCs and in Th cells. The percentage of OCRs broader than 500 bp are calculated. **(H)** Histogram showing distances of expression-concordant OCRs to transcription start sites (TSSs) of their neighboring genes in ILC and in Th. Percentage of OCRs within 1 kb of TSS is calculated. For box plots, the three horizontal lines of the box represent the third quartile, median, and first quartile, respectively, from top to bottom. The whiskers below and above the box show 5 and 95 percentiles. Statistical significance of peak size and distance of OCRs to their associated TSSs are calculated by two-sided Mann–Whitney U test. P. value above 0.05 is considered not significant, ***P < 0.001. OCRs, open chromatin regions. BPM, bins per million mapped reads.

Gene expression levels often exhibited correlation with the accessibility of corresponding chromatin loci (35, 36). To further understand the transcriptional disparities between ILCs and Th cells, we compared the chromatin accessibility across the ILC and Th genesets using publicly available sequencing of DNase I hypersensitive sites (DNase-seq) data (GSE172358) (37) (**Figure 3A**). Through this analysis, we identified 3,292 opening chromatin regions (OCRs) across the ILC genesets and 4,293 OCRs across the Th geneset (within 50 kb to the transcriptional start site or TSS). The accessibility of these OCRs was then compared between ILCs and Th cells. OCRs that exhibited accessibility changes consistent with the expression changes of their associated genes when comparing ILCs and Th cells were referred to as expression-concordant OCRs (**Figures 3A, B**). Conversely, OCRs showing discord accessibility and expression changes between ILC and Th subsets were categorized as expression-non-concordant OCRs. As a result, we identified 1,022 concordant OCRs across the ILC genesets, which we termed as ILC concordant OCRs (**Figure 3C**). These OCRs corresponded to majority (79.5%) of the genes in the ILC geneset (**Figure 3D**). Additionally, we identified 2,270 ILC non-concordant OCRs, encompassing 94.2% of the genes in the ILC geneset (**Supplementary Figures 4A, B**). Similarly, we discovered 1,559 concordant OCRs across the Th genesets, named as Th concordant OCRs (**Figure 3E**). These OCRs accounted for 72.4% in the genes in the Th geneset (**Figure 3F**). We also identified 2,734 Th non-concordant OCRs, corresponding to 92.6% of the genes in the Th geneset (**Supplementary Figure 4C, D**). Similar to RNA-seq, Th subsets for DNase-seq are differentiated *in vitro* whereas ILC subsets are isolated *in vivo*. For eliminating the interference of the tissue environment and differentiation method, we validated chromatin accessibility of ILC concordant OCRs and Th concordant OCRs in ATAC-seq data of ILC and Th subsets *in vivo* (25). As we anticipated, most of ILC concordant OCRs are specifically opened in ILC subsets, whereas most of Th concordant OCRs are specifically opened in Th subsets *in vivo* (**Supplementary Figure 5**). This confirmed that the specific regulatory regions of ILC geneset and Th geneset are not affected by environmental factors.

Furthermore, we performed a detailed characterization of the ILC and Th concordant and non-concordant OCRs associated with the ILC and Th genesets. Notably, we observed that the peak widths of the ILC concordant OCRs in all ILC subsets were broader compared with the peak widths of the Th concordant OCRs in the Th subsets (**Figures 3C, E**). To statistically confirm this difference, we quantified the peak widths of the ILC and Th concordant and non-concordant OCRs (**Figure 3G**). The ILC concordant OCRs exhibited a significant increase in width compared with the Th concordant OCRs (**Figure 3G**). Conversely, the width of the ILC non-concordant OCRs showed some variation and even reduction when compared with the width of the Th non-concordant OCRs (**Supplementary Figure 4E**). Previous studies had suggested that wider OCRs might result from the merging of multiple accessible regions and were more likely to be located in the promoter and super-enhancer regions of the associated genes (38). Therefore, we also investigated the genomic distribution of these ILC and Th concordant and non-concordant OCRs. Consistent with the previous studies (39–41), ILC concordant OCRs demonstrated a preferential distribution within 1 kilobase pair (kb) around TSSs compared with the Th concordant OCRs (**Figure 3H**). However, the distribution of ILC non-concordant OCRs did not show such a tendency (**Supplementary Figure 4F**). Overall, these findings suggest that the transcriptional regulation of the ILC geneset tends to heavily rely on promoter regions compared with the transcriptional regulation of the Th geneset.

### Different transcription factors are involved in distinguishing the functionalities of ILCs from Th cells

**Figure 4:**
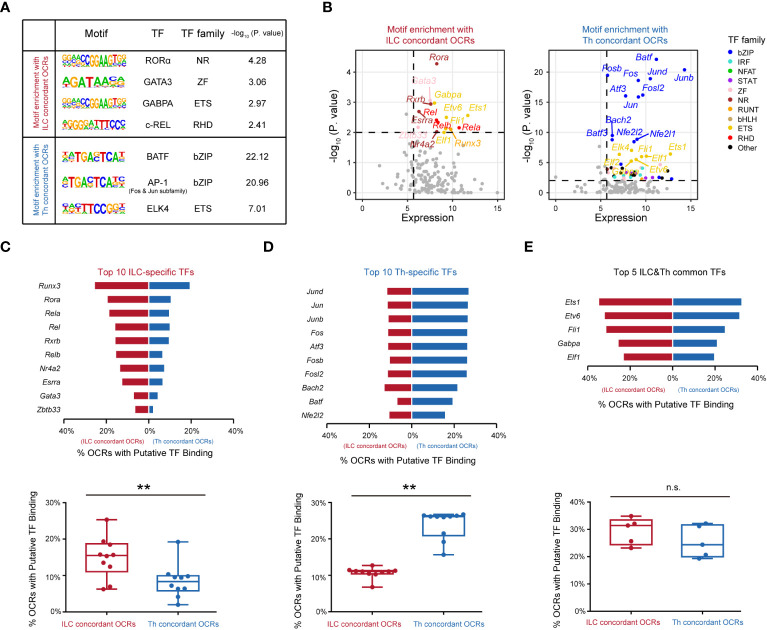
Distinct transcription factors are involved in distinguishing the functionalities of ILCs from Th cells. **(A)** Motif enrichment of expression-concordant OCRs in ILCs and in Th cells. The top significantly enriched motifs and the associated P. value are shown. TF and the TF family are annotated in the HOCOMOCO database and Homer software. Similar transcription factors in the same TF family are not shown. **(B)** Scatter plot of potential transcription factors that may bind to expression-concordant OCRs in ILCs and in Th cells. TF expression (X axis) and enrichment score (Y axis) are shown. Potential TFs in ILCs and Th cells are identified by TPM >50 in ILCs or Th cells, and P. value < 0.01. Colors indicate TF families as in (4A). **(C)** Bar chart showing percentage of OCRs with binding motifs of the top 10 ILC-specific TFs within the ILC concordant OCRs and within the Th concordant OCRs (top). The percentages of OCRs with binding motifs of the top 10 ILC-specific TFs within the ILC concordant OCRs and within the Th concordant OCRs are analyzed by the two-sided Wilcoxon test (bottom). **(D)** Bar chart showing percentage of OCRs with binding motifs of the top 10 putative TFs in Th cells, within the ILC concordant OCRs and within the Th concordant OCRs (top). The average percentages of OCRs with binding motifs of the top 10 Th-specific TFs within the ILC concordant OCRs and within the Th concordant OCRs are analyzed by the two-sided Wilcoxon test (bottom). **(E)** Bar chart showing percentage of OCRs with binding motifs of the top five ILC and Th common TFs, within the ILC concordant OCRs and within the Th concordant OCRs (top). The top 10 TFs in Th cells (TPM >50 in all three Th subsets) are ordered by P. value. The percentages of OCRs with binding motifs of the top five ILCs and Th common TFs within the ILC concordant OCRs and within the Th concordant OCRs are analyzed by two-sided Wilcoxon test (bottom). For box plots, the three horizontal lines of the box represent the third quartile, median, and first quartile, respectively, from top to bottom. The whiskers below and above the box show the 5th and 95th percentile. P. value above 0.05 is considered not significant, **P < 0.01. ns, no significance.

The observed differences in chromatin landscapes between the ILC and Th genesets suggested that they might be regulated by distinct transcriptional control mechanisms mediated by unique regulators. To explore this further, we conducted an analysis to identify enriched transcription factor binding motifs within the ILC and Th concordant OCRs. Indeed, we found that the ILC concordant OCRs displayed enriched binding motifs for transcription factors including RORα, GATA3, GABPA, and c-Rel (**Figure 4A**), whereas differentially enriched binding motifs were observed for transcription factors such as BATF, AP-1, and ELK4 in the Th concordant OCRs (**Figure 4A**). Subsequently, we considered the average expression levels of these predicted transcription factors across ILC or Th subsets, providing further evidence of their potential roles in driving the divergence of gene expression programs between ILCs and Th cells (**Figure 4B**). Importantly, the motifs for BATF and AP-1, both belonging to the bZIP family, were notably enriched in the Th concordant OCRs (**Figures 4A, B**). This observation aligned with the notion that these transcription factors could form the AP-1-BATF transcriptional complex, exerting crucial regulatory functions downstream of TCR signaling to promote the activation and function of Th cells (16, 42). In contrast, the transcriptional regulation of the ILC geneset appeared to rely on a distinct set of transcription factors, primarily belonging to different transcription factor families rather than the bZIP family (**Figure 4B**). On the other hand, a diverse range of transcription factors were enriched in the expression-non-concordant OCRs in both ILCs and Th cells, indicating their unlikely involvement in regulating the gene expression in the ILC or Th geneset (**Supplementary Figures 6A, B**).

In our analysis of the ILC concordant OCRs, we identified 10 potential transcription factors with highly enriched binding motifs and significant expression levels, namely, RORα, GATA3, RXRβ, c-REL, REL-B, REL-A, ERRα, ZBTB33, NR4A2, and RUNX3. Notably, RORα, a member of the nuclear receptor family, displayed the highest enrichment in the ILC concordant OCRs. This finding aligned with previous studies that highlighted the crucial roles of RORα in the development, maintenance, and immune activation of ILC2s and ILC3s (43–45). Similarly, we observed significant enrichment of GATA3 binding motifs, supporting the notion that GATA3 played a regulatory role in the development, expansion, and activation of both ILC2s and ILC3s in our previous studies (12, 46, 47). Thus, these results validated the accuracy of our analysis. Furthermore, we observed substantial enrichment of binding motifs for REL-A, REL-B, and c-Rel in the ILC concordant OCRs. Their transcription factors, belonging to the RHD family, formed complexes with the NF-κB subunits (p50 for REL-A and c-Rel, and p52 for REL-B) (48). This suggested a potential involvement of NF-κB in the transcriptional regulation of the ILC geneset. In contrast, in the Th concordant OCRs, all of the top 10 enriched potential transcription factors with significant expression levels belonged to the bZIP family. This highlighted the critical role of AP-1 in regulating T-cell function under TCR signaling (49). Additionally, we discovered substantial enrichment of potential transcription factors from the ETS family in both the ILC and Th concordant OCRs. For example, ETS1 was identified as occupying a significant number of expression-concordant OCRs in both cell types. Based on literature, ETS1 had been reported to play crucial regulatory roles in both the expansion of ILC2s and the activation of Th cells (50, 51). Overall, our analysis identified the top 10 highly enriched potential transcription factors in ILCs or Th cells based on their expression-concordant OCRs, as well as five potential transcription factors that were commonly enriched in both cell types.

Subsequently, we investigated the frequencies of binding motifs associated with these potential transcription factors in the expression-concordant OCRs of ILCs and Th cells. The top 10 potential transcription factors specific to ILCs displayed enhanced regulation of the ILC geneset in ILCs compared with the Th geneset in Th cells, indicating their importance in distinguishing the functionalities of ILC and Th cells (**Figure 4C**). Moreover, the top 10 Th-specific transcription factors from the bZIP family exhibited significant regulation of the Th geneset specifically in Th cells, whereas their impact on the ILC geneset in ILCs was relatively limited (**Figure 4D**). This underscored the essential role of AP-1 in distinguishing the functionalities of Th cells from ILCs. Additionally, the five potential transcription factors commonly enriched in the expression-concordant OCRs of ILCs and Th cells demonstrated comparable regulation of the ILC geneset in ILCs and the Th geneset in Th cells (**Figure 4E**). However, these transcription factors regulated a relatively high frequency of genes within both the ILC and Th genesets, suggesting their critical roles. In contrast, the distribution patterns of these potential transcription factors in the expression-non-concordant OCRs of ILCs and Th cells did not align with the gene expression patterns (**Supplementary Figures 6C–E**). Collectively, these findings indicate that distinct functionalities of ILCs and Th cells are regulated by different transcription factors.

### Similar effector roles of ILCs and Th cells are operated by distinct regulatory machineries

**Figure 5:**
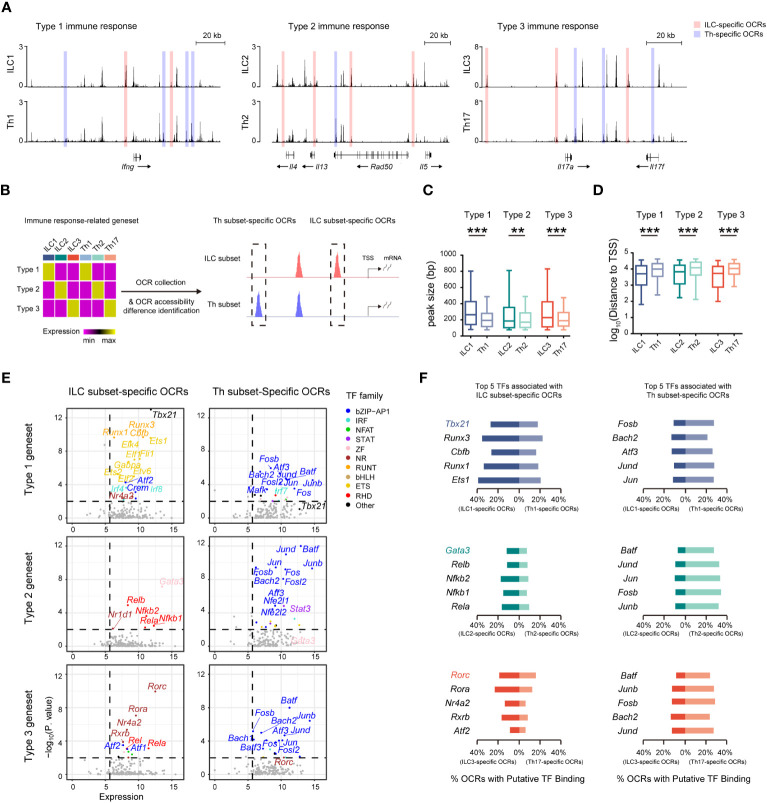
Similar effector roles of ILCs and Th cells are operated by distinct regulatory machineries. **(A)** DNase-seq tracks at the *Ifng* locus, *Il4*, *Il13*, and *Il5* loci, and *Il17a* and *Il17f* loci in the indicated ILC and Th subsets. ILC-specific OCRs in corresponding ILC and Th subsets are defined as, for example, accessibility ILC1/Th1 >1.5, minimum accessibility of ILC1 > maximum accessibility of Th1, and minimum accessibility of ILC1 >0.5 (chromatin accessibility of OCRs are calculated to BPM). Similarly, Th-specific OCRs are defined as, for example, accessibility Th1/ILC1 >1.5, minimum accessibility of Th1 > maximum accessibility of ILC1, and minimum accessibility of ILC1 >0.5. **(B)** Schematics of identifying ILC-specific OCRs and Th-specific OCRs associated with each respective immune response-specific geneset. **(C)** Box plot showing peak size of ILC-specific and Th-specific OCRs related to the type 1, type 2, and type 3 genesets. Statistical significance is calculated using two-sided Mann–Whitney U test. **(D)** Box plot showing distances of ILC-specific and Th-specific OCRs related to the type 1, type 2, and type 3 genesets to TSSs of neighboring genes. Statistical significance is calculated using two-sided Mann–Whitney U test. **(E)** Scatter plot of potential transcription factors that may bind to ILC-specific OCRs or Th-specific OCRs related to the type 1, type 2, and type 3 genesets. Expression levels of TFs (X axis) and their enrichment score (Y axis) are shown. Potential TFs in ILCs versus Th cells are identified by TPM >50 in corresponding ILC and Th subsets, and P. value <0.01. Colors indicate TF families as in **Figure 4A**. **(F)** Bar chart showing percentage of putative binding of OCRs by the top five TFs enriched in ILCs and Th cells within OCRs associated with the type 1, type 2, or type 3 geneset. For box plots, the three horizontal lines of the box represent the third quartile, median, and first quartile, respectively, from top to bottom. The whiskers below and above the box show the 5th and 95th percentiles. A P. value above 0.05 is considered not significant, **P < 0.01, ***P < 0.001.

Given the presence of distinct regulatory machineries for the ILC and Th genesets, we further wondered whether the immune response-related genesets, which exhibited similar expression in both ILC subsets and their corresponding Th subsets, were differentially regulated. Remarkably, we found that chromatin regions of signature effector genes associated with different immune responses, such as the *Ifng* locus in ILC1s and Th1 cells, the *Il4*, *Il5*, and *Il13* loci in ILC2s and Th2 cells, and the *Il17a* and *Il17f* loci in ILC3s and Th17 cells, all displayed variant accessibility between ILC subsets and their corresponding Th subsets (**Figure 5A**). Therefore, we defined the ILC and Th subset-specific OCRs based on their differential chromatin accessibility between ILC subsets and their corresponding Th subsets (**Figure 5B**, **Supplementary Figure 7A**). ILC3- and Th17-specific OCRs displayed exclusive chromatin accessibility in ILC3 and Th17 subsets *in vivo*, respectively (**Supplementary Figure 7B**). In addition, ILC2- and Th2-specific OCRs displayed higher accessibility in ILC2 and Th2 subsets *in vivo*, respectively (**Supplementary Figure 7C**). Notably, the overall width of ILC subset-specific OCRs exceeded that of the corresponding Th subset-specific OCRs, with the former preferentially located in close proximity to TSSs, suggesting distinct regulation of these genesets between the two cell types (**Figures 5C, D**, **Supplementary Figures 7D, E**). Consequently, we conducted an analysis of potential transcription factors inferred from these subset-specific OCRs in ILCs and Th cells. As expected, different sets of potential transcription factors with significant expression were enriched by the subset-specific OCRs in ILC subsets and the corresponding Th subsets, respectively (**Figure 5E**). Interestingly, these potential transcription factors exhibited considerable consistency with those we inferred from the expression-concordant OCRs to the ILC or Th genesets, indicating the utilization of the same regulatory machinery to govern effector functions in each subset. Notably, the master transcription factors *Tbx21*, *Gata3*, and *Rorc* displayed the highest enrichment by the ILC subset-specific OCRs, underscoring their indispensable regulatory roles in the effector functions of ILC subsets. Additionally, upon assessing the distribution patterns of binding motifs linked to these potential transcription factors in the ILC or Th subset-specific OCRs, we confirmed the preferential binding of potential transcription factors specific to ILC subsets to the ILC subset-specific OCRs, whereas those specific to Th subsets tended to interact with the Th subset-specific OCRs (**Figure 5F**). Therefore, despite the striking similarity in effector roles between ILC subsets and their corresponding Th subsets, the underlying regulatory mechanisms remain distinctive.

## Discussion

ILCs are often regarded as the innate counterparts to Th cells in the adaptive immune system due to their shared functionalities and regulatory mechanisms (3, 31). However, it is important to recognize the fundamental differences between these two cell types as innate and adaptive lymphocytes, respectively. In this study, we have demonstrated the presence of significant distinctions in functionalities and underlying regulatory mechanisms between ILCs and Th cells by conducting integrative transcriptome and chromatin landscape analyses.

Bulk RNA-seq has been widely used as a mature approach for transcriptome analysis for many years. More recently, single-cell transcriptome analysis, or scRNA-seq, has emerged as a highly valuable tool in the field (52, 53). While scRNA-seq offers several advantages over bulk RNA-seq, it also comes with certain limitations (54). One noticeable drawback is the higher cost associated with scRNA-seq, resulting in smaller sample sizes for each cell type. In contrast, bulk RNA-seq is comparatively more cost-effective, and thus researchers can easily access numerous publicly available datasets, particularly for the ILC and Th transcriptomes that are relevant to our study (55). Furthermore, due to technological differences, scRNA-seq typically captures fewer genes compared with bulk RNA-seq (52). In our study, we sought to harness the advantages of both technologies. Therefore, we compiled and processed a collection of 294 publicly available bulk RNA-seq datasets for ILCs and Th cells, which were subsequently analyzed using algorithms designed for scRNA-seq. Through this integrated approach, we successfully identified two genesets specific to all ILCs or Th cells, as well as three genesets specific to different types of immune responses. Although for bulk RNA-seq data, most of Th cell datasets are derived from *in vitro* differentiated lineages, and most ILCs are isolated from mucosal tissue *in vivo*, we observed that our defined ILC geneset and Th geneset still represent difference between ILC and Th subsets *in vivo*, suggesting that these genesets represent fundamental distinctions between ILC and Th cells.

The accuracy and reliability of the genesets we defined are ensured by their generation from a large number of bulk RNA-seq datasets derived from various experiments conducted in different laboratories. This comprehensive approach guarantees that the genesets should accurately capture the fundamental differences between ILCs and Th cells, as well as between different types of immune responses. Consequently, we performed GO enrichment analysis to gain further insights into the functional disparities underlying the transcriptional differences. While there are some shared features between the ILC and Th genesets, they are predominantly enriched in distinct pathways. Consistent with our understanding of T-cell expansion following activation, we observed a preferential enrichment of cell cycle-related pathways in the Th geneset. On the other hand, the ILC geneset exhibited specific enrichment in pathways associated with their role in maintaining tissue homeostasis. Moreover, despite ILC and Th subsets being involved in diverse immune responses, we identified a similarity in pathway enrichment for immune response-related genesets, indicating the existence of certain fundamental similarities in their T helper cell functions through cytokine secretion.

The differential enrichment of pathways in the ILC and Th genesets suggests that the functionalities of these two cell types may be regulated by distinct mechanisms. Notably, the expression-concordant OCRs associated with the ILC geneset are generally broader in width compared with those associated with the Th genesets. ILC concordant regions preferentially localize around TSSs, which correspond to the promoter regions of genes. Interestingly, the OCRs specific to the immune response-related genesets in ILCs also exhibit a similar distribution pattern as compared with distribution of the Th-specific OCRs in Th cells. This characteristic may confer an advantage for multiple transcription factors to bind to the promoter regions in ILCs, thereby facilitating rapid transcription initiation upon cell activation.

We have conducted further analysis on the expression-associated OCRs for the ILC and Th genesets, as well as on the ILC- and Th-specific OCRs for the immune response-related genesets, to infer the potential involvement of transcription factors. It is noteworthy that Th cells exhibit a heightened tendency to specifically utilize AP-1-mediated regulation, which occurs downstream of TCR signaling. In contrast, ILCs do not favor AP-1 and exhibit enhanced utilization of master transcription factors specific to their respective subsets. Additionally, our findings indicate a preferential reliance on NF-κB for regulatory processes in ILCs. Furthermore, despite the similar effector functions of ILC subsets and their corresponding Th subsets, the underlying regulatory mechanisms are also largely distinct, aligning with their innate and adaptive lymphocyte properties, respectively (56). Consequently, our study provides valuable insights into the functional and regulatory differences between ILCs and Th cells, contributing to a comprehensive understanding of their unique roles during immune responses.

## Materials and methods

### Data acquisition

Raw data for gene expression profiles (RNA-seq) of ILC and Th subsets were retrieved from Sequence Read Archive (SRA); accession numbers are given in **Figure 1A** and **Supplementary Table 1**. Raw data for RNA-seq of ILC and Th subsets *in vivo* were retrieved from SRA under accession numbers SRP060453, SRP069783, and SRP337230. Chromatin accessibility profiles (DNase-seq) of ILC and Th subsets were retrieved from SRP315389, and ATAC-seq of ILC and Th subsets *in vivo* were retrieved from SRP069783.

### RNA-seq data processing

The RNA-seq reads were aligned to the GRCm38/mm10 assembly of mouse genome using HISAT2 (v 2.2.1), and quantified by featureCounts. The gene expression level was counted by featureCounts (v 2.0.3) against mouse GRCm38 genome assembly (v 94). Transcripts per million mapped reads (TPM) were calculated using R package scuttle (v1.4.0). Genes with significantly high expression levels were filtered by TPM >10 in at least one ILC or Th subset. Batch effect removal was performed by the “removeBatchEffect” function in the limma package (v3.50.3) (57). Dimension reduction of the gene expression matrix before or after batch effect removal by the Uniform Manifold Approximation and Projection (UMAP) algorithm was performed by R package Seurat (v4.3.0) (58). “ScaleData”, “RunPCA”, and “RunUMAP” were sequentially executed, and top20 principle components (PCs) were used for UMAP analysis. GSEA was performed by GSEA software (v3.0).

### Differential expression analysis and gene ontology enrichment

For differential expression analysis between ILCs and Th cells and among three types of immune responses, the FindConservedMarkers function in the Seurat package was performed (minimum of log_2_ fold change >0.25 and maximum of P. value <0.01). The FindMarkers function was used in differential expression analysis in each ILC and Th subsets (log_2_ fold-change >0.25 and P value < 0.01). GO analysis was performed by overrepresentation test with R package clusterProfiler (v4.2.2). Function “compareCluster” was performed for GO enrichment of ILC - and Th-specific genesets (q value < 0.05), and the top 100 GO terms in q value are shown in enrichment map by the “emapplot” function in the “enrichplot” package. Function enrichGO was performed for GO enrichment of immune response-specific genesets.

### DNase-seq data processing

DNase-seq reads were mapped to the mm10 genome with Bowtie2 (v2.4.4). Non-redundant reads with MAPQ ≥10 were remained. The remaining reads were sorted using Samtools (v 1.13). DNase-seq peaks (OCRs) were called by MACS2 (v2.2.7.1) with settings of –nomodel –extsize 75, based on a q-value threshold of 0.01. DNase-seq reads in each OCR were quantified using bedtools (v2.27.1). Bins per million mapped reads (BPM) values of OCRs were calculated with R-package scuttle as TPM in RNA-seq data.

### Peak annotation and differential OCR analysis

Annotation of OCRs to their neighboring genes were performed by the annotatePeak function in R package ChIPseeker (v1.30.3). OCRs within 50 kb to TSS of neighboring genes were defined as OCRs related to these genes. For OCRs related to genes in the ILC and Th genesets, concordant OCRs were defined as OCRs exhibiting concordant accessibility changes with the expression changes of their related genes; the residual OCRs were defined as non-concordant OCRs. For example, in OCRs related to the ILC geneset, the ILC concordant OCRs were defined as OCRs with a fold change of ILC/Th >1.5, minimum BPM in both repeats of each ILC subset > maximum BPM in both repeats of each Th subset, and BPM of both repeats in all ILC subsets >0.5. The residual OCRs related to the ILC geneset are defined as ILC non-concordant OCRs. Similar criteria are used to define Th concordant and non-concordant OCRs.

For OCRs related to immune response-related genesets, ILC-specific OCRs are defined as OCRs with fold change between ILC subsets and the corresponding Th subsets >1.5, minimum BPM in both repeats of the ILC subset > maximum BPM in both repeats of the corresponding Th subset, and BPM of both repeats in the ILC subset >0.5. A similar criterion is used to define Th concordant OCRs.

### Motif enrichment

Transcription factor motif enrichment was performed by the findMotifsGenome function in Homer software (v4.10), using the HOCOMOCO database and the database included in HOMER. For each OCR, transcription factor binding sites were annotated by FIMO software in MEME Suite (v5.0.5), using a p-value threshold of 0.0001.

### Data visualization and statistics

Data were analyzed by R version 4.1.2. Bar charts, pie charts, box plots, scatter plots, and histograms were operated by ggplot2 (v3.4.2). Heatmaps in schematic illustration were performed by pheatmap (v1.0.12). Heatmaps of OCRs in DNase-seq were visualized by deepTools (v3.5.1). DNase-Seq tracks were visualized using UCSC Genome Browser. The statistical significance of GO enrichment and motif enrichment were calculated by a two-sided hypergeometric test. P values above 0.05 were considered not significant, *P < 0.05 **P < 0.01, ***P < 0.001.

## Data availability statement

The original contributions presented in the study are included in the article/**Supplementary Material**. Further inquiries can be directed to the corresponding author.

## Author contributions

YZ: Conceptualization, Data curation, Formal Analysis, Investigation, Methodology, Software, Visualization, Writing – original draft. LH: Data curation, Formal Analysis, Methodology, Software, Visualization, Writing – original draft. GR: Data curation, Methodology, Writing – original draft. YYZ: Data curation, Methodology, Writing – original draft. XZ: Data curation, Methodology, Writing – original draft. CZ: Conceptualization, Funding acquisition, Methodology, Project administration, Supervision, Writing – review & editing.
