# Supplementary material for: Distinct regulatory machineries underlying divergent chromatin landscapes distinguish innate lymphoid cells from T helper cells
Source: Front Immunol. 2023 Dec 1;14:1271879. doi: 10.3389/fimmu.2023.1271879 (PMC10722145; doi:10.3389/fimmu.2023.1271879)
Supplement: Supplementary file 1 [file Image_1.pdf]

Supplementary data for

Distinct Regulatory Machineries Underlying Divergent Chromatin Landscapes Distinguish Innate Lymphoid Cells from T Helper Cells

Yime Zhang[a,b,1], Luni Hu[a,b,1], Guanqun Ren[a,b], Yanyu Zeng[a,b], Xingyu Zhao[a,b], Chao Zhong[a,b,c,d,*]

[a] *Institute of Systems Biomedicine, School of Basic Medical Sciences, Peking University Health Science Center, Beijing 100191, China;*

[b] *Beijing Key Laboratory of Tumor Systems Biology, Peking University, Beijing 100191, China;*

[c] *Department of Immunology, School of Basic Medical Sciences, Peking University Health Science Center, Beijing 100191, China;*

[d] *NHC Key Laboratory of Medical Immunology, Peking University, Beijing 100191, China.*

[*] Corresponding author.

E-mail address: zhongc@pku.edu.cn (C. Zhong).

[1] Contributed equally to this work

Figures S1 to S7

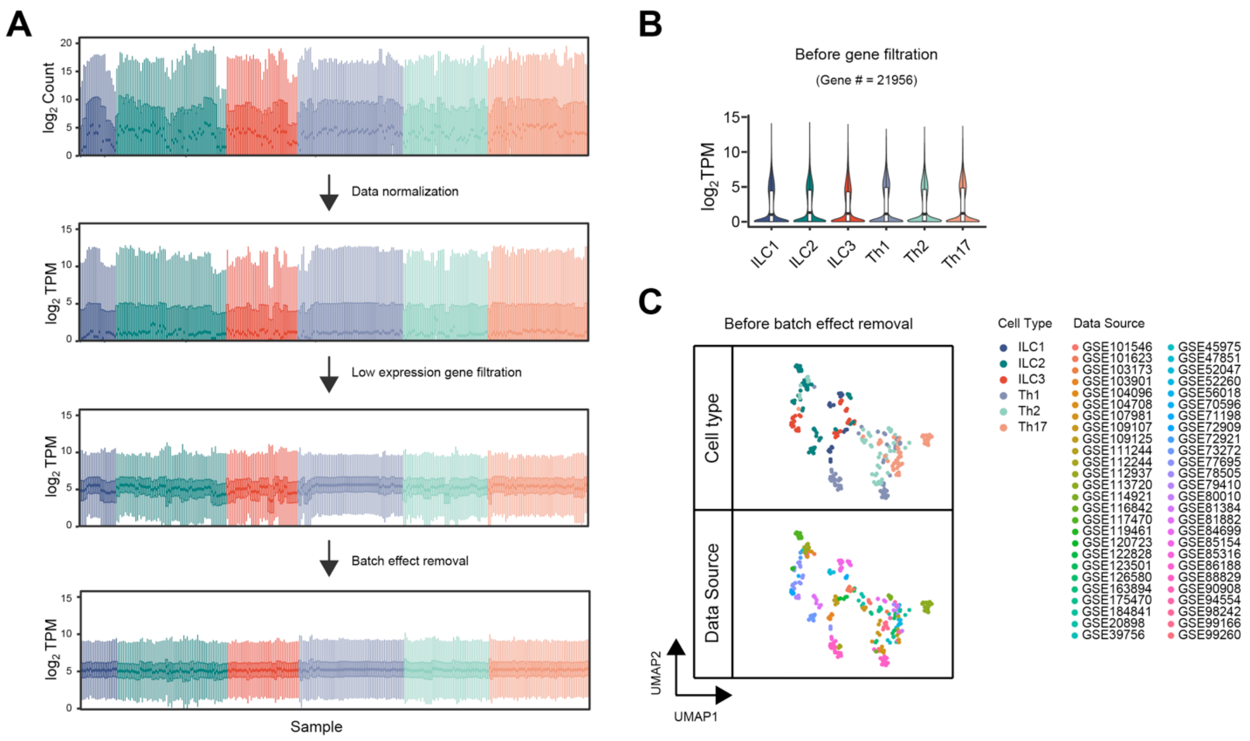

Fig. S1. Procedure of integrated bulk RNA-seq analysis between ILC and Th subsets. **A**: Violin plot showing average gene expression ($\log_2$ TPM) in each cell type before filtering genes with low expression levels. **B**: UMAP plot showing distribution of ILC and Th cell bulk RNA-seq samples before batch effect removal. Cell types and data sources are annotated. **C**: Box plot showing gene expression in each bulk RNA-seq sample throughout data preprocessing procedure. Log_2 Count represents gene expression before normalization, while $\log_2$ TPM represents gene expression after normalization, low expression gene filtration and batch effect removal. UMAP, Uniform Manifold Approximation and Projection. TPM, transcripts per million mapped reads.

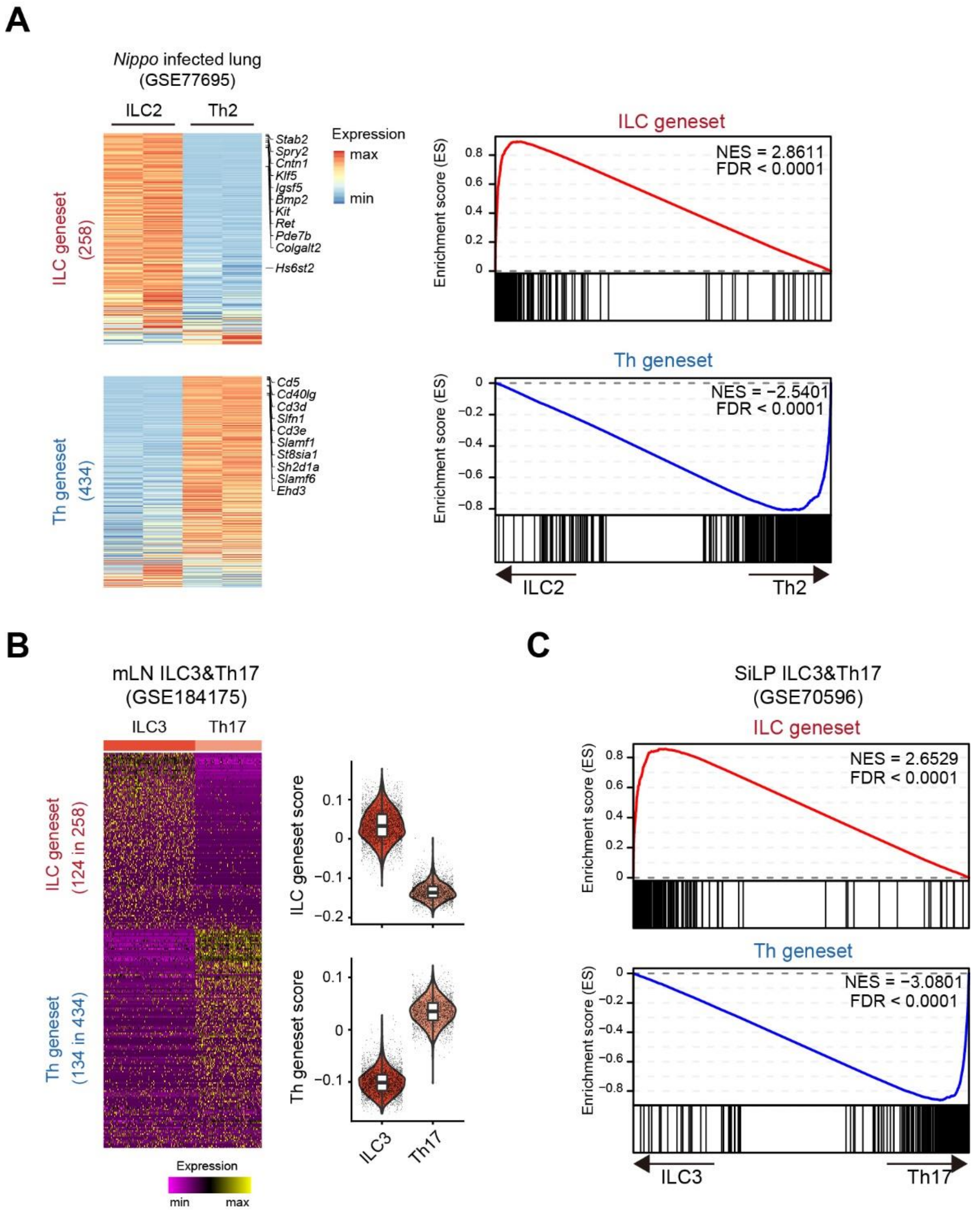

Fig. S2. ILC geneset and Th geneset are minimally affected by environmental factors. **A**: Heatmap and Gene set enrichment analysis (GSEA) showing expression of ILC-specific genes and Th-specific genes in lung ILC2 and Th2 from infecting mice with *Nippostrongylus brasiliensis* (GSE77695). **B**: Heatmap and violin plot showing expression of ILC-specific genes and Th-specific genes in ILC3 and Th17 from mesenteric lymph nodes (GSE184175). **C**: GSEA showing expression of ILC-specific genes and Th-specific genes in ILC3 and Th17 from small intestine (GSE70596).

Fig. S3

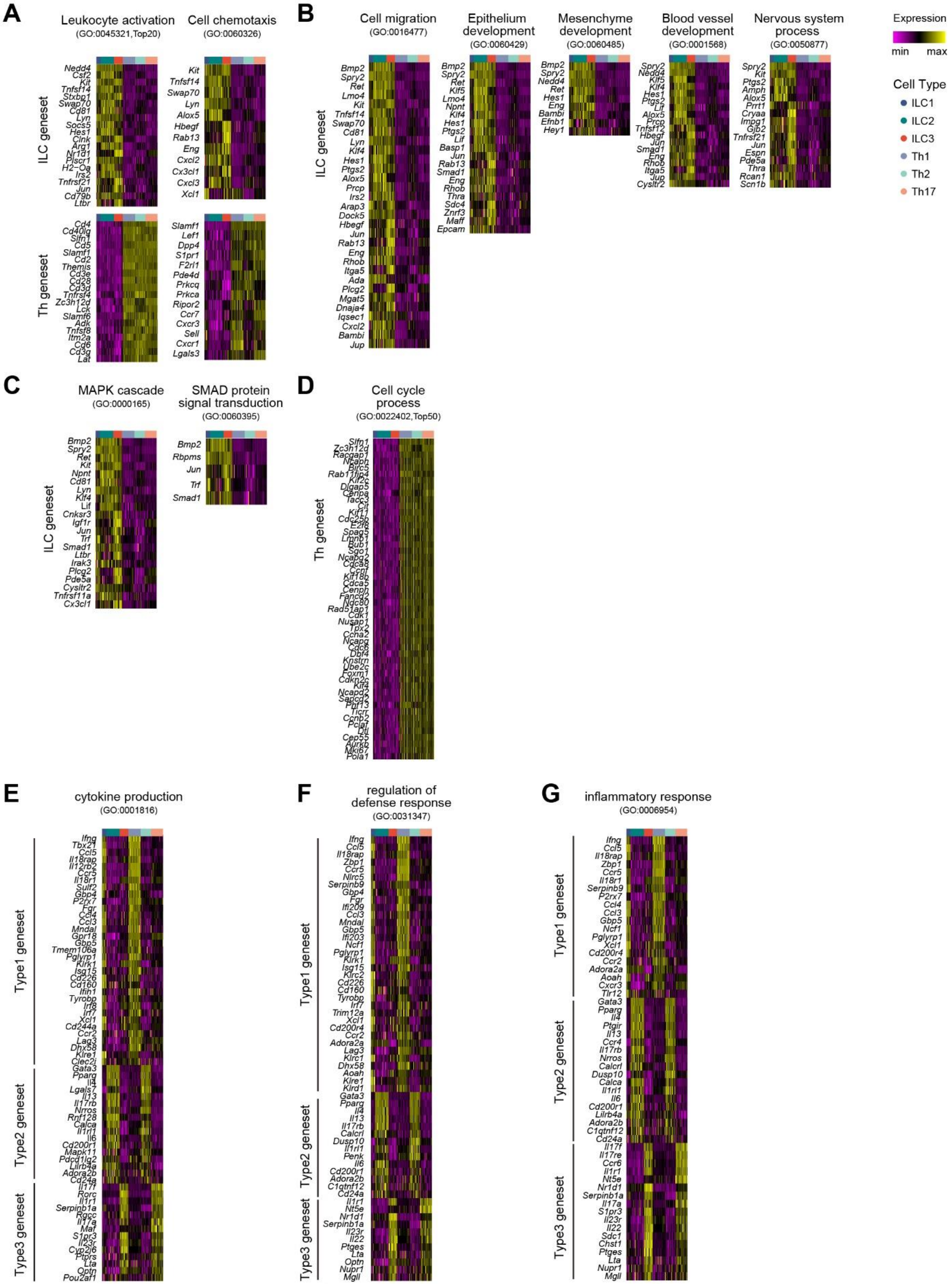

A
Leukocyte activation
(GO:0045321,Top20)
Cell chemotaxis
(GO:0060326)
ILC geneset
Th geneset
B
Cell migration
(GO:0016477)
Epithelium development
(GO:0060429)
Mesenchyme development
(GO:0060485)
Blood vessel development
(GO:0001568)
Nervous system process
(GO:0050877)
Expression
min
max
Cell Type
ILC1
ILC2
ILC3
Th1
Th2
Th17
C
MAPK cascade
(GO:0000165)
SMAD protein signal transduction
(GO:0060395)
D
Cell cycle process
(GO:0022402,Top50)
E
cytokine production
(GO:0001816)
F
regulation of defense response
(GO:0031347)
G
inflammatory response
(GO:0006954)
Type1 geneset
Type2 geneset
Type3 geneset

Fig. S3. Profiling of ILC- and Th-specific genes enriched in each functional pathway. **A**: Heatmap showing ILC-specific genes in “leukocyte activation” pathway and Th-specific genes in “cell chemotaxis” pathways. The top 20 genes in each pathway are ordered by fold changes between ILC and Th subsets. **B** and **C**: Heatmap showing ILC-specific genes in the indicated pathways enriched in the ILC geneset. **D**: Heatmap showing the top 50 Th-specific genes enriched in “cell cycle process”. The genes are ordered by fold changes between ILC and Th subsets. **E** - **G**: Heatmap showing the specific genes in the indicated pathways enriched in the immune response-specific genesets .

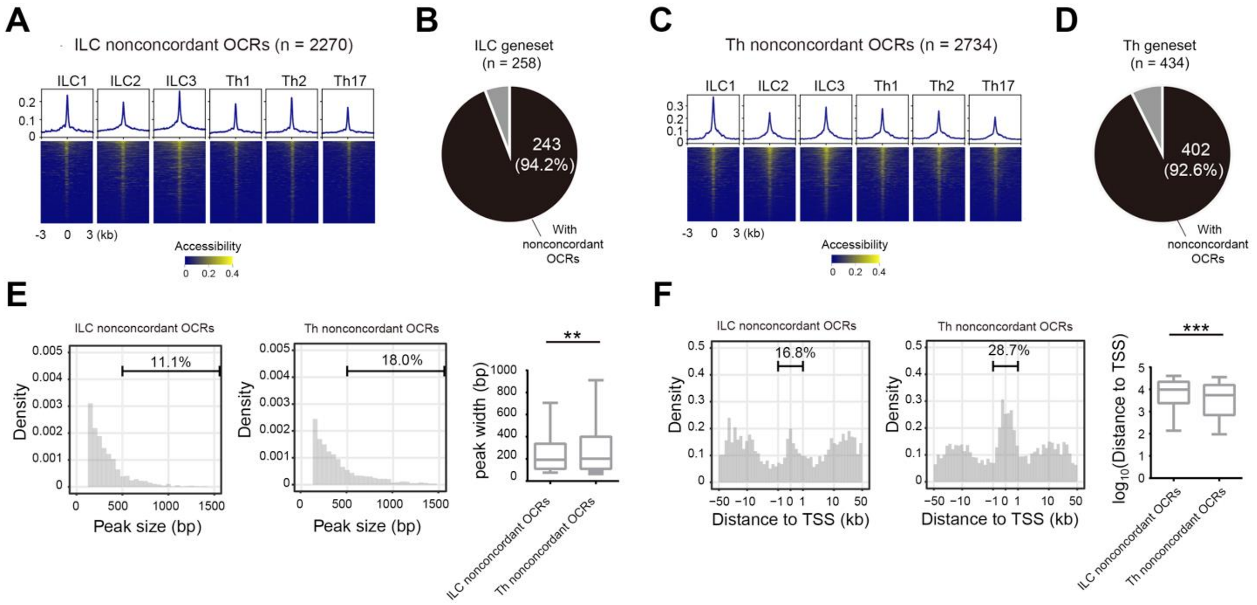

Fig. S4. Comparison of peak size and genome distribution of expression-nonconcordant OCRs between ILC and Th subsets. **A**: Heatmap showing chromatin accessibility of ILC nonconcordant OCRs across ILC and Th subsets. Profile plot illustrates average chromatin accessibility of the corresponding regions. **B**: Pie chart showing the number and proportion of genes in the ILC geneset with ILC nonconcordant OCRs at their gene loci. **C**: Heatmap showing chromatin accessibility of Th nonconcordant OCRs across ILC and Th subsets. Profile plot illustrates average chromatin accessibility of the corresponding regions. **D**: Pie chart showing the number and proportion of genes in the Th geneset with Th nonconcordant OCRs at their gene loci. **E**: Histogram showing peak size distribution of expression-nonconcordant OCRs in ILCs and in Th cells. Percentage of OCRs with peak width broader than 500bp is calculated. **F**: Histogram showing distances of expression-nonconcordant OCRs in ILCs and Th cells to their related TSSs. Percentage of OCRs located within 1kb to TSSs is calculated. For box plots, the three horizontal lines of the box represent the third quartile, median and first quartile, respectively, from top to bottom. The whiskers below and above the box show 5 and 95 percentile. Statistical significance of peak size and distance of OCRs to their associated TSSs are calculated by two-sided Mann-Whitney U test. P. value above 0.05 is considered not significant, *P < 0.05 **P < 0.01, ***P < 0.001. OCRs, open chromatin regions. BPM, bins per million mapped reads.

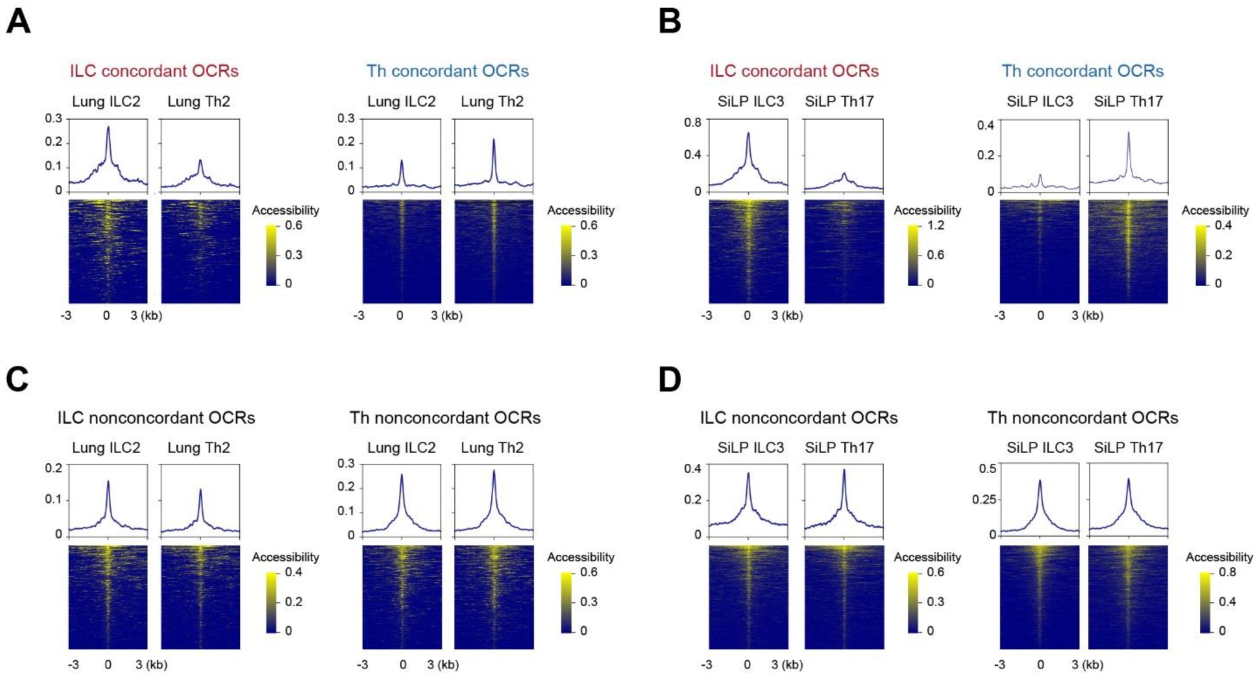

Fig. S5. Expression-concordant opening chromatin regions are minimally affected by environmental factors. **A**: Heatmap showing chromatin accessibility of ILC concordant OCRs and Th concordant OCRs in lung ILC2 and Th2 isolated from infecting mice with *Nippostrongylus brasiliensis*. Profile plot illustrates average chromatin accessibility of the corresponding regions. **B**: Heatmap showing chromatin accessibility of ILC concordant OCRs and Th concordant OCRs in ILC3 and Th17 isolated from lamina propria of small intestine. Th17 cells are isolated from IL-17 reporter mice. Profile plot illustrates average chromatin accessibility of the corresponding regions. **C**: Heatmap and profile plot showing chromatin accessibility of ILC nonconcordant OCRs and Th nonconcordant OCRs in lung ILC2 and Th2 isolated from infecting mice with *Nippostrongylus brasiliensis*. **D**: Heatmap and profile plot showing chromatin accessibility of ILC nonconcordant OCRs and Th nonconcordant OCRs in ILC3 and Th17 isolated from lamina propria of small intestine. All of ATAC-seq data above are accessed from GSE77695.

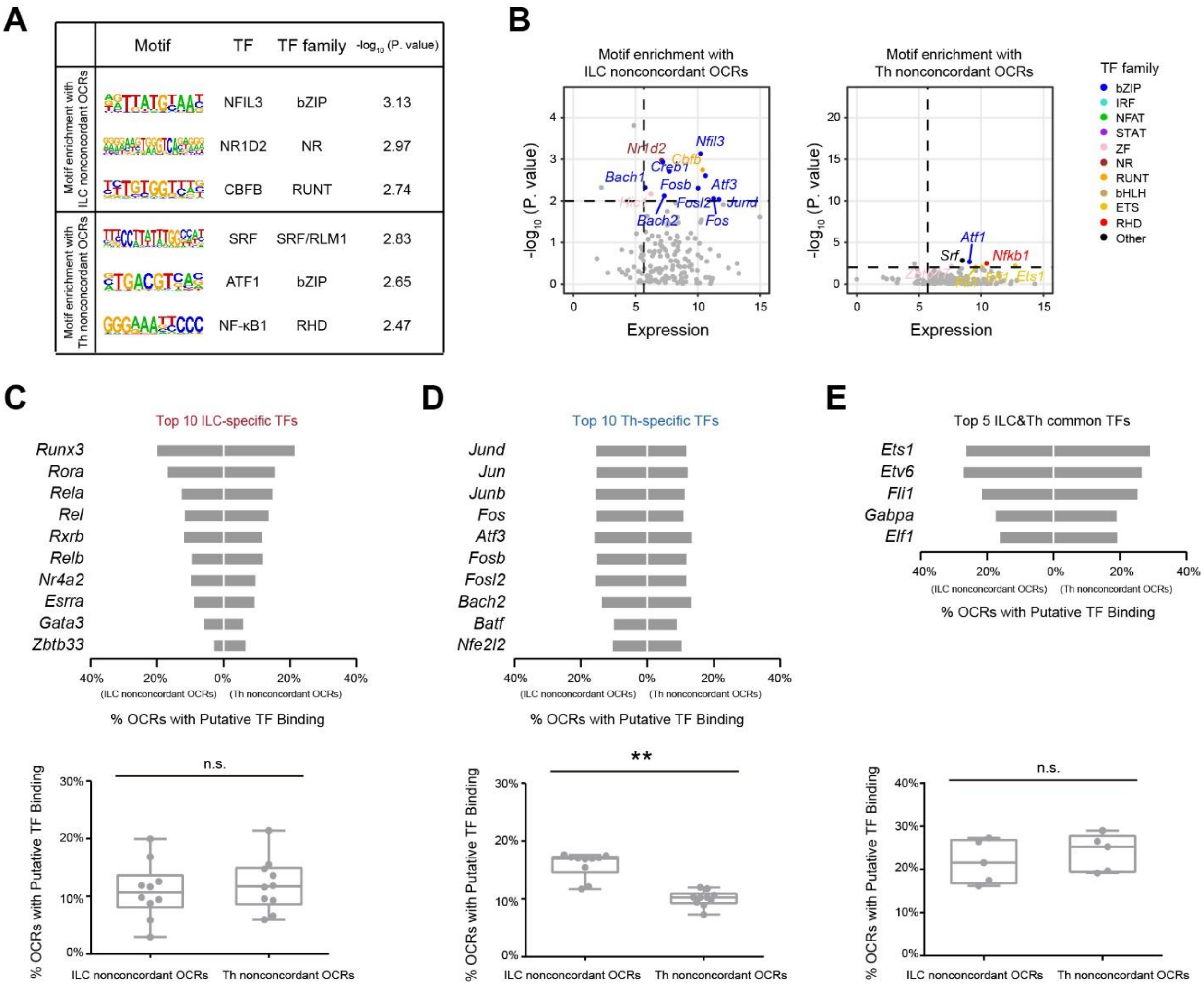

Fig. S6. Distinct transcriptional regulation of ILC and Th geneset *via* expression-concordant OCRs. **A**: Motif enrichment of expression-nonconcordant OCRs in ILCs and Th cells. The top significantly enriched motifs and the associated P. value are shown. TF and TF family are annotated based on HOCOMOCO database and Homer software. Similar transcription factors in the same TF family are not shown. **B**: Scatter plot of potential transcription factors that may regulate expression-nonconcordant OCRs in ILCs and Th cells. Expression levels of transcription factors (X axis) and their enrichment score (Y axis) are shown. Potential transcription factors in ILCs and Th cells are identified by TPM > 50 in all three ILC or Th subsets, and P. value < 0.01. **C**: Bar chart showing percentage of OCRs with binding motifs of the top10 ILC-specific TFs, within the ILC

nonconcordant OCRs and within the Th nonconcordant OCRs (top). The percentages of OCRs with binding motifs of the top 10 ILC-specific TFs within the ILC nonconcordant OCRs and within the Th nonconcordant OCRs are analyzed by two-sided Wilcoxon test (down). **D**: Bar chart showing percentage of OCRs with binding motifs of the top10 Th-specific TFs, within the ILC nonconcordant OCRs and within the Th nonconcordant OCRs (top). The percentages of OCRs with binding motifs of the top 10 Th-specific TFs within the ILC nonconcordant OCRs and within the Th nonconcordant OCRs are analyzed by two-sided Wilcoxon test (down). **E**: Bar chart showing percentage of OCRs with binding motifs of the top 5 ILC and Th common TFs, within the ILC nonconcordant OCRs and within the Th nonconcordant OCRs (top). The percentages of OCRs with binding motifs of the top 5 ILC and Th common TFs within the ILC nonconcordant OCRs and within the Th nonconcordant OCRs are analyzed by two-sided Wilcoxon test (down). For box plots, the three horizontal lines of the box represent the third quartile, median and first quartile, respectively, from top to bottom. The whiskers below and above the box show 5 and 95 percentile. P. value above 0.05 is considered not significant, *P < 0.05 **P < 0.01, ***P < 0.001. ns, no significance

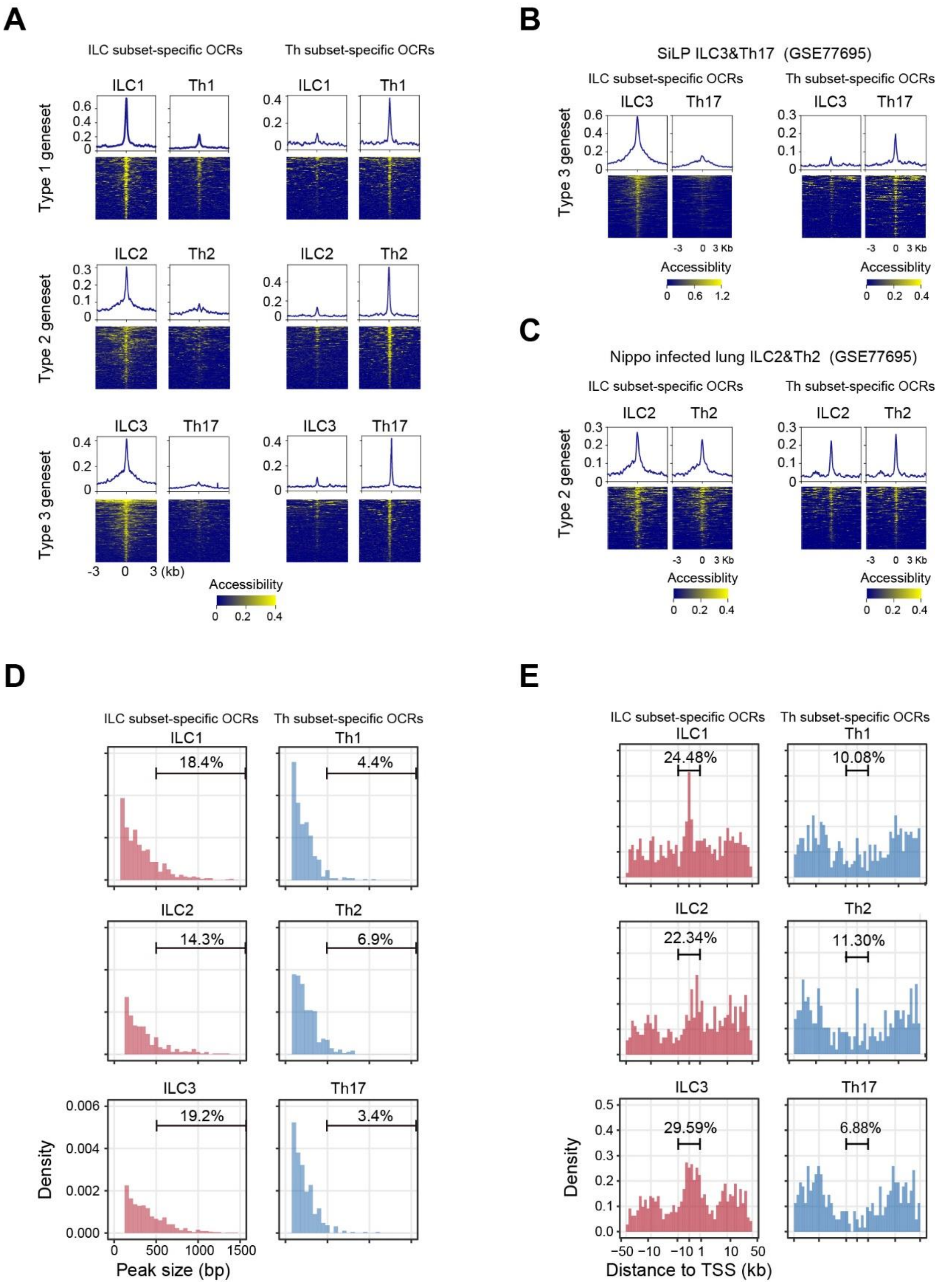

Fig. S7. ILC- and Th-specific OCRs associated with immune response-specific genesets possess different properties. **A**: Heatmap showing chromatin accessibility of ILC- and Th-

specific OCRs associated with type1, type 2, and type 3 genesets in the corresponding ILC or Th subset. Profile plot illustrating the average chromatin accessibility of corresponding regions. **B**: Heatmap showing chromatin accessibility of ILC3- and Th17-specific OCRs in ILC3 and Th17 isolated from lamina propria of small intestine. Th17 cells are isolated from IL-17 reporter mice. Profile plot illustrates average chromatin accessibility of the corresponding regions. **C**: Heatmap showing chromatin accessibility of ILC2- and Th2- specific OCRs in lung ILC2 and Th2 isolated from infecting mice with *Nippostrongylus brasiliensis*. Profile plot illustrates average chromatin accessibility of the corresponding regions. **D**: Histogram showing peak size distribution of ILC specific- and Th specific-OCRs associated with type1, type 2, and type 3 genesets. Percentage of OCRs with peak width broader than 500bp is calculated. **E**: Histogram showing distances of ILC specific- and Th specific-OCRs associated with type1, type 2, and type 3 genesets to their related TSSs. Percentage of OCRs located within 1kb to TSSs is calculated. For box plots, the three horizontal lines of the box represent the third quartile, median and first quartile, respectively, from top to bottom. The whiskers below and above the box show 5 and 95 percentile. Statistical significance of peak size and distance of OCRs to their associated TSSs are calculated by two-sided Mann-Whitney U test. P. value above 0.05 is considered not significant, *P < 0.05 **P < 0.01, ***P < 0.001.
